# Improved many-objective particle swarm optimization based welding sequence optimization research

**DOI:** 10.1371/journal.pone.0343554

**Published:** 2026-03-05

**Authors:** Lei Dong, Shimin Gu, Jianwei Dong, Qiukai Ji, Jinfeng Liu

**Affiliations:** 1 Shenyang Institute of Computing Technology Chinese Academy of Sciences, Shenyang, China; 2 University of Chinese Academy of Sciences, Beijing, China; 3 AVIC Information Technology Co., Ltd., Beijing, China; 4 School of Mechanical Engineering Jiangsu University of Science and Technology, Zhenjiang, Jiangsu, China; Islamic Azad University Mashhad Branch, IRAN, ISLAMIC REPUBLIC OF

## Abstract

Welding sequence optimization (WSO) for ship components is a complex, multi-objective, and nonlinear challenge. Traditional methods relying heavily on engineer experience often lead to inadequate decisions, resulting in excessive deformation, residual stress, and even cracking. To address this, we propose a systematic WSO method for ship structural parts that integrates both process and geometric constraints. The optimization objectives are formally defined through objective functions quantifying structural deformation and residual stress. For solving this high-dimensional problem, an Improved Many-Objective Particle Swarm Optimization (IMaOPSO) algorithm is developed. IMaOPSO enhances the classical PSO by incorporating an adaptive fuzzy dominance relation to improve selection pressure and a perturbation term guided by elite solutions to maintain population diversity. This ensures rapid convergence to a well-distributed set of high-quality solutions. Simulation analysis of different welding sequences is conducted based on the SYSWELD software platform. A case study on a ship deck structure demonstrates that IMaOPSO outperforms several established algorithms (NSGA-II, SPEA2, SMPSO) in convergence speed and stability. The optimal sequence identified reduces average deformation by 32.6% to 62.2% compared to other methods, confirming the proposed method’s significant practical engineering value for improving welding quality and efficiency in shipbuilding.

## 1. Introduction

The shipbuilding industry in China has achieved remarkable progress, with its shipbuilding output reaching a leading global position as early as 2010. This growth is part of a broader national strategy to transition from a manufacturing giant to a leader in advanced, intelligent manufacturing. The “14th Five-Year Plan” explicitly emphasizes enhancing the intelligent degree of ship construction, particularly through the introduction of intelligent manufacturing technologies to improve product quality and manufacturing capabilities. However, the industry concurrently faces significant challenges, including insufficient scientific and technological innovation capacity, weak research in key common technologies, and reliance on external sources for critical components and supporting product technologies [1,2].

**Fig 1:**
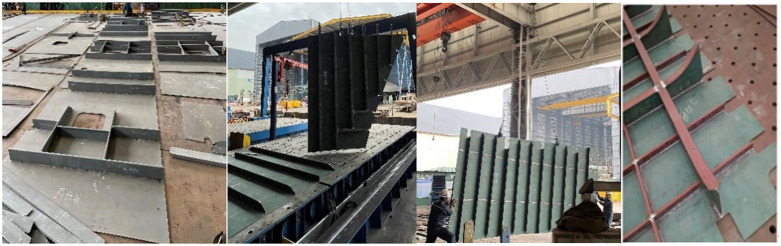
Common complex thin plate components.

Welding, particularly submerged arc welding, is one of the most critical and time-consuming processes in shipbuilding, accounting for 60% to 80% of the total production time. A single 50,000-ton vessel can involve the assembly of approximately 2,000 complex sheet metal components. These components exhibit considerable diversity in geometry, dimensions, thicknesses, and structural configurations. While the general external hull forms may be consistent, the internal structures vary substantially, as illustrated by the common complex thin-plate components shown in Fig 1. Common complex thin plate components.

In current industrial practice, the determination of the welding sequence relies heavily on the extensive experience of engineers. While valuable, this empirical approach lacks adaptability and quantitative analysis when dealing with the complexity and variability inherent in modern ship components. This often leads to suboptimal decisions, resulting in excessive welding deformation, residual stress, and even cracking—issues that compromise structural integrity, increase rectification costs, and delay production schedules.

Although intelligent planning of welding sequences has seen successful applications in domains like automotive body-in-white assembly, its research and application in the specific context of ship welding remain limited. The unique scale, complexity, and stringent performance requirements of ship structures present distinct challenges that general-purpose optimization methods may not address effectively.

To bridge this gap, this paper takes a complex ship-component structure as the research object. We formulate a Welding Sequence Optimization (WSO) problem with structural deformation and residual stress as the primary optimization objectives, subject to process constraints such as welding direction, method, and weld length. An Improved Many-Objective Particle Swarm Optimization (IMaOPSO) algorithm is developed and integrated with Finite Element Analysis (FEA) to predict deformation and residual stress, thereby solving the complex ship welding sequence planning problem efficiently.

**Fig 2:**
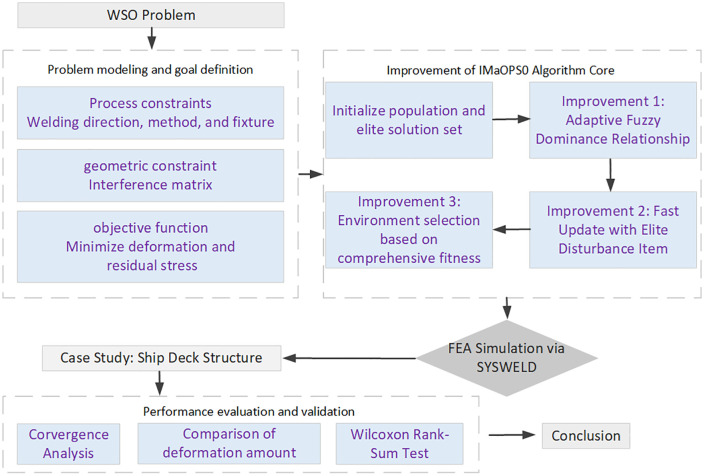
Research flowchart.

The remainder of this paper is organized as follows. Section 2 reviews the relevant research background and existing literature on welding sequence optimization. Section 3 details the modeling of the welding sequence optimization problem and the establishment of the objective function. Section 4 presents the proposed IMaOPSO algorithm. Section 5 provides experimental results and discussion based on a case study. Finally, Section 6 concludes the research and suggests directions for future work. The process of this article is shown in Fig 2.

## 2. Research background

Welding sequence optimization (WSP) is the sequencing of the welding direction and welding order of each part of the product, which is a key part of the welding process in the mechanical and marine industries and can strongly influence the deformation and residual stress of the welded structure. By selecting the appropriate welding optimization sequence, on the one hand, the welding deformation can be effectively controlled and the welding quality can be guaranteed. On the other hand, it can reduce the production cost and improve the production efficiency of the products.

**Fig 3:**
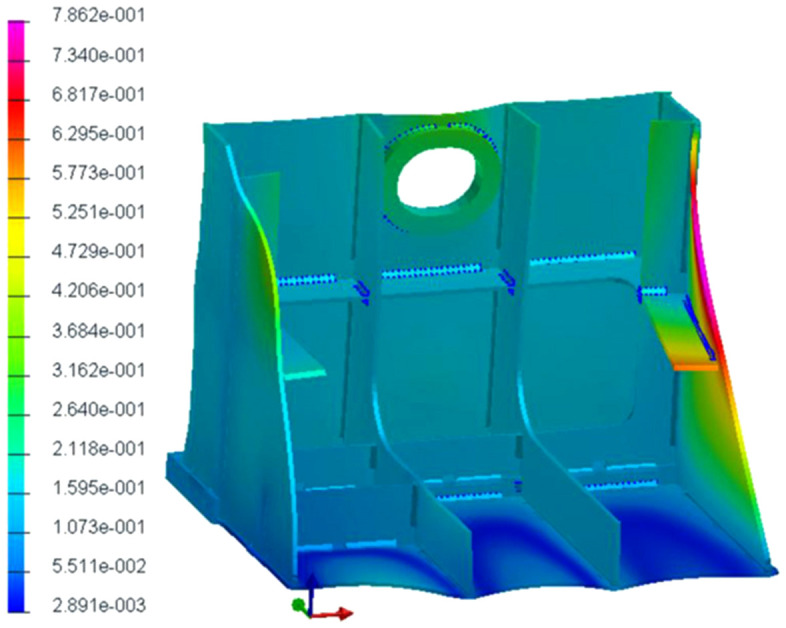
Finite element model of ship components.

When studying different welding sequences to obtain the best geometry for ship grouping, it’s not practical to create a physical sample by testing every possible welding sequence because of the high costs, long time – consuming nature, and high complexity involved. Therefore, the experiments are replaced by using FEA computational simulation tools (SYSWELD) as well as intelligent optimization algorithms. As shown in the case of Fig 3.

The research on optimization of product welding sequences can be traced back to the 1940s. Since the 1990s, artificial intelligence methods have been applied to welding sequence optimization. Fukuda et al. [3] put forward a method grounded in neural network theory as an efficient means to optimize the welding time and sequence. This method takes into account parameters like the distance the welding torch travels, shrinkage, and welding symmetry. The study was carried out experimentally on a simple symmetric structure. Marian et al. [4] and Wang [5] used genetic algorithm and ant colony algorithm to solve the WSP problem, respectively; Gao et al. [6] proposed an artificial immune system combining immunity and clone selection to study the WSP problem; Lv et al. [7] proposed a discrete particle swarm optimization algorithm to tackle the welding sequencing problem. It is founded on the mapping strategy, the encoding method for particle position and velocity, and the particle update operation; Wang et al. [8] and Li [9] proposed a hybrid algorithm of chaotic particle swarm algorithm and discrete particle swarm algorithm to solve the WSP problem, correspondingly; Zhang et al. [10] utilized the immune algorithm, which is capable of surmounting the premature convergence issue inherent in the particle – swarm optimization algorithm, to address the WSP; Ghandi et al. [11] studied the topology, structure and complexity of the search space for the assembly sequence planning (ASP) and proposed a breakout local search (BLS) algorithm to obtain high-quality ASP solutions based on the characteristic that most of the local optimal assembly sequences are uniformly distributed.

Tabar et al. [12] applied digital twin technology to the optimization of assembly welding sequences. By proposing an optimization method of spot-welding sequence to ensure the quality of spot-welding geometry. In the digital twin with guaranteed geometric accuracy, assembly parameters are chosen for each individual assembly unit, and the optimal welding sequence is ascertained from the perspective of time. This method reduces the processing time of spot welding by 30%.

In the research on welding deformation control, numerical simulation and finite element analysis are classic methods for revealing the deformation mechanism and conducting virtual verification. Woo et al. [13] systematically analyzed the welding sequence of ship outer plates to control deformation based on finite element numerical simulations such as intrinsic strain and interface elements. Lostado et al. [14] comprehensively used the finite element method, genetic algorithm, and regression tree to obtain the temperature field and deformation amount through the thermal cloud map of the machining simulation model. Marques et al. [15] clearly proposed to conduct virtual detection of the welding process through numerical simulation, providing data support for the finite element model and verifying the accuracy of the simulation and experimental results. Tao Ma [16] established the welding trajectory equation and heat source model, and compared four segmented welding sequences of pipes with different wall thicknesses through finite element analysis.

To overcome the problems of high cost and low optimization efficiency in traditional simulation calculations, various intelligent optimization algorithms have been introduced into the field of welding sequence optimization. Wang [17] optimized both the welding path length and the total deformation amount through an improved multi – objective particle swarm algorithm. Shao et al. [18] used the discrete particle swarm optimization algorithm to optimize the welding sequence and direction, significantly reducing the residual deformation and strain. Danning Fan [19] proposed an optimization method combining the ConvLSTM - UNet neural network and the NSGA – II genetic algorithm to address the time – consuming issue of finite element simulation. Yuan Mingxin [20] constructed a welding sequence optimization model aiming at minimizing deformation and proposed an improved immune clone optimization algorithm for solving it.

Furthermore, the development of neural networks and hybrid algorithm frameworks provides more powerful tools for welding deformation prediction and sequence optimization. Wu Chun biao [21] focused on developing an optimization framework that couples an artificial neural network and a swarm intelligence algorithm to specifically reduce the welding deformation of thin – walled square tubes. Hao Xu [22] constructed a dataset using welding numerical simulation and experiments and employed a simulated annealing – optimized BP neural network model to determine the optimal welding sequence, overcoming the defect that the traditional BP network is prone to falling into local optima.

In the specific aspects of engineering application and process innovation, the research focuses on improving the quality and efficiency of specific joint forms and assembly processes. Haipeng Miao [23] proposed a sequence optimization method and process improvement strategy for segmented multi – layer and multi – pass welding for the automatic welding of thick – plate arc – shaped groove special – shaped line welds. Pan et al. [24] proposed an automatic non – linear loading sequence planning method, used a part layout map with attributes for semantic modeling, and combined heuristic graph search and an improved particle swarm algorithm to generate high – quality welding sequences. Roham [25] focused on the assembly stage, evaluated the welding sequence using the finite element influence factor method, and proposed a detection method that can bypass the spring – back calculation, significantly shortening the optimized operation time.

In addition to the aforementioned classical methods, a series of advanced nature-inspired multi-objective optimization algorithms have emerged in recent years, such as the multi-objective moose herd optimization, many-objective cheetah optimizer, and adaptive predator–prey algorithm [26–28], which have demonstrated potential in addressing complex engineering problems. Meanwhile, hybrid strategies integrating surrogate models, decision-maker methods (e.g., RSM-VIKOR), or domain-specific approaches (e.g., distribution network optimization, structural design) have also achieved significant progress [29–32]. These studies provide valuable references and inspiration for the design of an efficient and robust many-objective particle swarm optimization algorithm for welding sequence optimization in this work.

## 3. Modeling of welding sequence optimization problem and establishment of the objective function

During ship construction, the ship is usually divided into several segments and each segment is built separately. During the welding of a single segment, the welding units in the segment can be divided into component level, small group standing level, medium group standing level, and large group standing level according to the similarity of the components. In a single segment, the number of component levels is large, which largely affects the welding speed, so it is not reasonable to consider the welding sequence of the segment from a component-level perspective. The complexity of the problem can be significantly simplified by using the group stand-up level as the base building block.

In this paper, the welding process is analyzed from the team stage to the middle assembly, the structural deformation and residual stress during welding are studied, and the optimal welding sequence is achieved by minimizing the deformation and stress. Process constraints such as welding direction, welding method, and weld length are considered during the welding process, and the effect of each constraint on deformation, strain, completion time, and weld continuity is analyzed.

### 3.1 Process constraints analysis

According to the process constraints such as welding direction, welding method, and welding tool in the actual welding process of ship segments, the influence of each constraint on structural deformation and residual stress is analyzed.

(1) Welding method and efficiency

The places where the assemblies touch each other are connected by welding, and the length of each weld can be obtained by the ship’s computer-aided design software. Before welding assembly i, check the number of components that need to be welded between the welded assembly and the to-be-welded assembly i. The welding process is carried out in the welding sequence, and the total time required for the assembly in the welding process is


$$S_{wm}=\sum_{i=1}^{n}S_{w_{i}}$$ (1)

Here, i is the group to be welded and n denotes the quantity of groups that are in contact with the group to be welded. If different welding methods exist at the connection between two assemblies, they can be transformed into the same type of welding according to the relationship of welding rates.

(2) Welding direction

During the welding process, the overall length of the weld is constant, but different welding directions can have an impact on the structural deformation by geometric interference phenomena during the welding process. Therefore, the interference matrix serves to characterize the geometric constraints across all welding directions, so the interference matrix can be described as:


$$IM_{\text{m}}\text{=}\left[\begin{array}{cccc} @cccc@I_{11m} & I_{12m} & ... & I_{1nm}\\ I_{21m} & I_{22m} & ... & I_{2nm}\\ ... & ... & ... & ...\\ I_{n1m} & I_{n2m} & ... & I_{mnm} \end{array}\right]$$ (2)

Here, m∈{±x,±y,±z} indicates welding direction; *I*_*ijm*_ is a binary variable, If member *i* interferes with member *j* in the m-welding direction, then *I*_*ijm*_ = 1, otherwise *I*_*ijm*_ = 0. The primary role of the interference matrix lies in determining the subsequent weld member i along with its corresponding welding direction.

(3) Welding tool

Different auxiliary tools are needed in the welding process of products, and different tools are used for welding construction [33]. Therefore, welding tools are often changed during the welding process of products, which increases the welding time and reduces the welding efficiency. Unnecessary tool replacement should be avoided. The welding tool of each component is defined as $T_{i}(i=1,2,3,4,5)$.

### 3.2 Objective function

Ship segments are affected by different process parameters during the welding process and the main consideration is the numerical variation of structural deformation and residual stresses during the welding process. The objective of the optimization process is to obtain the best welding sequence that satisfies the conditions. To gradually investigate the influence of various process parameters upon the optimization objective, on this basis, the multi-objective optimization function for the optimization of the welding sequence are presented as below.


$$\left\{ \begin{array}{c} @l@minF\left(x\right)=min\left(F_{\text{1}}\left(x\right),F_{\text{2}}\left(x\right)\right)\\ =minF_{\text{1}}\left\{U_{\text{1}}\left(x\right),S_{\text{1}}\left(x\right),\sigma_{\text{1}}\left(x\right)\right\},F_{\text{2}}\left\{U_{\text{1}}\left(x\right),S_{\text{1}}\left(x\right),\sigma_{\text{1}}\left(x\right)\right\}\\ x\in \{x_{\text{1}},x_{\text{2}},x_{\text{3}},......\} \end{array}\right.$$ (3)

Here, $x\in \{x_{\text{1}},x_{\text{2}},x_{\text{3}},......\}$ indicates weld seam; $F_{\text{1}}\left(x\right)$ and $F_{\text{2}}\left(x\right)$ denote the residual stress and the total weld deflection, respectively.

## 4. IMaOPSO based welding sequence optimization

The particle swarm optimization (PSO) algorithm, as an efficient swarm intelligence optimization method, has been widely applied to various engineering optimization problems, such as the optimal design of structural dampers [34–36] and the improvement of seismic performance [37–38]. These studies indicate that the PSO algorithm exhibits good robustness and scalability when solving engineering problems with complex constraints and multi-objective characteristics. This section first introduces the basic principles of classical PSO and then elaborates on the improved strategies proposed in this study for the welding sequence optimization problem.。

### 4.1 Classical particle swarm optimization

The particle swarm optimization algorithm is an evolutionary computational technique that was successively proposed and developed by Kennedy and Eberhart [39] in 1995 as a representation of the movement of a programmed organism in a school of fish or a flock of birds and was initially used for the evolutionary emulation of social conduct. The traditional particle swarm algorithm simulates the social behavior of birds searching for food. Every single particle adjusts its location within the population by tracking the best – fitting solution of the particle and that of the population according to its position, speed, and fitness function, and iterates to obtain the optimal solution. The advantage of this algorithm is that it is simple to implement and requires fewer parameters to be adjusted.

Assume that there exists a particle swarm composed of M particles, and the quantity of optimization variables is D. The search space of this particle swarm is D – dimensional. The particle swarm algorithm makes use of the value of the fitness blocking function as the discriminant, and each particle has its own position and velocity properties, representing one possible solution of the fitness function. Update the particle’s individual optimal solution $P_{best}^{t}$ and the global optimal solution $G_{best}^{t}$ in each iteration, and each generation of particles search and move under the D-dimensional space, tracking the current particle to get two fitness values. The velocity and position of the particle are adjusted in accordance with Eq. (1) and Eq. (2).


$$v_{i}^{t+1}=\omega v_{i}^{t}+c_{\text{1}}r_{\text{1}}(p_{i}^{t}-x_{i}^{t})+c_{\text{2}}r_{\text{2}}(p_{g}^{t}-x_{i}^{t-1})$$ (4)

$$x_{i}^{t-1}=x_{i}^{t}+v_{i}^{t+1}$$ (5)

Here, t is the number of iterations, ω is the inertia weight, c1 and c2 are learning factors, and r1 and r2 are random numbers within [0,1]. $x_{i}^{t}$ is the position of particle i in t iterations, $v_{i}^{t}$ is the position of the individual optimal solution of particle i after t iterations, and $p_{g}^{t}$ is the position of the global optimal solution after t iterations. The particle velocity update equation consists of three parts. $v_{i}^{t}$ indicates the current velocity state of the particle, $r_{\text{1}}(p_{i}^{t}-x_{i}^{t})$ embodies the next iteration velocity update from the self-learning ability, and $r_{\text{2}}(p_{g}^{t}-x_{i}^{t-1})$ indicates the next iteration velocity update from the information sharing among some population particles.

### 4.2 Improved many-objective optimization particle swarm optimization

The actual welding sequence design is a multi-objective optimization problem, and conventional multi-objective optimization algorithms can effectively solve multi-objective optimization problems with 2 or 3 objectives, but the results of solving high-dimensional multi-objective optimization problems (n ≥ 4) are not satisfactory, and the main reasons exist, With the growth of problem objective dimensions increases, the use of conventional Pareto dominance relations will potentially lead to insufficient pressure on the algorithm to select non-dominated solutions, making it difficult for non-dominated solutions to effectively approximate the true Pareto frontier. In addition, since the Pareto optimal frontier of the high-dimensional multi-objective optimization problem is a hypersurface, it leads to an exponential increase in the size of the Pareto optimal solution set and the corresponding feasible solution search space, which results in a significant decrease in the convergence speed of the algorithm.

When the standard particle swarm algorithm is used to deal with the welding sequence optimization problem, the shortcomings of poor diversity and slow convergence often occur. An adaptive fuzzy dominance relation is proposed and applied to a high-order dimensional multi-objective particle swarm algorithm. This dominance relationship adaptively adjusts the fuzzy membership dominance threshold with the step size amplitude, which not only improves the dominance probability among individuals but also achieves fine regulation of the population pressure of the high-dimensional multi-objective particle swarm optimization algorithm, so as to better promote the convergence of the algorithm. The comparison between the two algorithms is shown in Table 1.

**Table 1:** Comparison of two algorithms.

Improvement dimension	Standard MOPSO	IMaOPSO	Improvement purpose and effect
1. Dominance Relation	Pareto Dominance	Adaptive Fuzzy Dominance Relation	Solve the problem of insufficient selection pressure in high-dimensional objective spaces. By adaptively adjusting the fuzzy membership dominance threshold, sufficient selection pressure can still be provided when the number of objective dimensions increases, driving the population to converge towards the Pareto front.
2. Velocity Update	Guided by Pbest and Gbest	Elite-guided Perturbation Term	Solve the problem of premature convergence and maintain population diversity. Introduce neighboring particles selected from the elite solution set as a perturbation term to guide the particles to explore unknown regions and effectively escape from local optima.
3. Environmental Selection	Crowding Distance	Fitness-based Selection	Explicitly balance convergence and diversity. Use a comprehensive fitness function (which combines dominance degree and crowding degree) to replace the single crowding distance, so as to more accurately select the next-generation population with both good convergence and distribution properties.

#### 4.2.1 Construction of the objective function.

In the field of welding sequence planning, the ideal welding sequence ought to be a viable sequence featuring the greatest welding efficiency and the lowest welding difficulty. In this paper, several factors with greater influence on the welding of assembled products are mainly considered, and an objective function with structural deformation, residual stress, completion time, and welding continuity as evaluation indicators is constructed, as shown in Eq. (6).


$$F=n_{r}+\omega_{\text{1}}S_{i}+\omega_{\text{2}}\sigma_{i}+\omega_{\text{3}}\frac{u_{d}}{N-2}+\omega_{\text{4}}\frac{t_{i}}{N-1}$$ (6)

Here, N is the number of components grouped in the composition. $n_{r}$ is the quantity of members that do not satisfy the geometric interference. $u_{d}$ is the number of welding direction changes. $t_{i}$ is the welding completion time. $S_{i}$ is the total amount of structural deformation during the welding process. $\sigma_{i}$ is the residual stress value. $\omega_{\text{1}}$, $\omega_{\text{2}}$, $\omega_{\text{3}}$ and $\omega_{\text{4}}$ are the weight coefficients of evaluation indexes respectively. 0 ≤ $\omega_{\text{1}}$, $\omega_{\text{2}}$, $\omega_{\text{3}}$, $\omega_{\text{4}}$ ≤ 1, and $\omega_{\text{1}}$+$\omega_{\text{2}}$+$\omega_{\text{3}}$+$\omega_{\text{4}}$=1. A, $\omega_{\text{1}}$, $\omega_{\text{2}}$, $\omega_{\text{3}}$ and $\omega_{\text{4}}$ are obtained from Eq. (7).


$$\omega_{k}=\frac{n+1-k}{\sum_{i=1}^{n}}=\frac{(n+1-k)*2}{(n+1)n}$$ (7)

Here, n is the quantity of evaluation criteria, here n = 4; k is the importance ranking of the evaluation criteria.

#### 4.2.2 Improved way of updating particle velocity.

**Fig 4:**
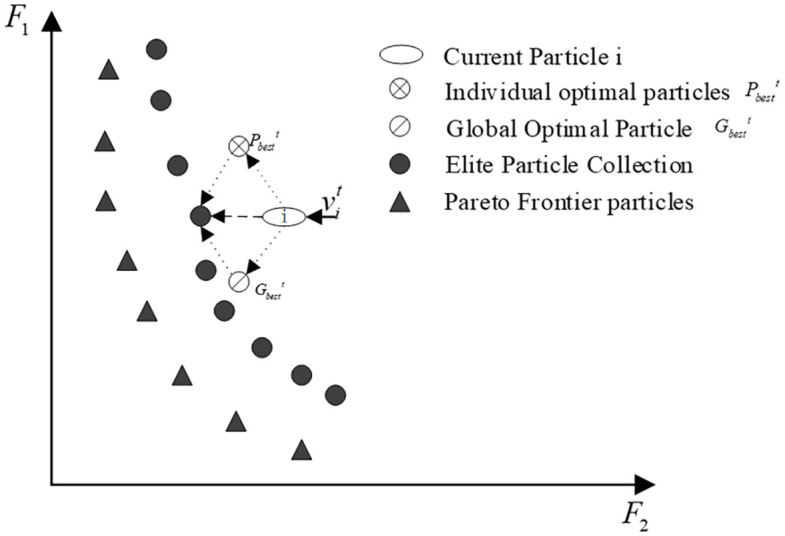
Diagram of particle velocity update in the classical multi-objective particle swarm algorithm.

The velocity of particle i in the classical particle swarm algorithm is updated following equation 4. It is easy to know that the change of the moving speed $v_{i}^{t}$ of particle i in the t-th generation is jointly affected by its individual historical optimal position $P_{best}^{t}$ and the global optimal position $G_{best}^{t}$. Fig 4 illustrates the current velocity trend of particle i as an example of a 2-objective optimization problem.

In the early stage of classical particle swarm algorithm, the particles fly faster and have a strong global search capability. Nevertheless, during the later phase of the algorithm, if the historical optimal position of the particle itself is relatively close to the global optimal position, it will easily lead to the aggregation of a large number of particles, making the multiplicity of the particles within the swarm worse, and may cause the algorithm to converge prematurely.

By considering a modification of the velocity equation of particles in the classical particle swarm algorithm, a perturbation term is added to enhance the multiplicity of the population, thus preventing the algorithm from getting trapped in a local extremum. where the perturbed particle is picked from the elite solution set to be the individual with the closest Euclidean distance from the current particle i. The particle velocity is updated by the improved particle velocity as shown in Eq. (8).


$$v_{i}^{t+1}=\omega v_{i}^{t}+c_{\text{1}}r_{\text{1}}(p_{i}^{t}-x_{i}^{t})+c_{\text{2}}r_{\text{2}}(p_{g}^{t}-x_{i}^{t-1})+c_{\text{3}}r_{\text{3}}(Pd_{i}^{t}-x_{i}^{t})$$ (8)

**Fig 5:**
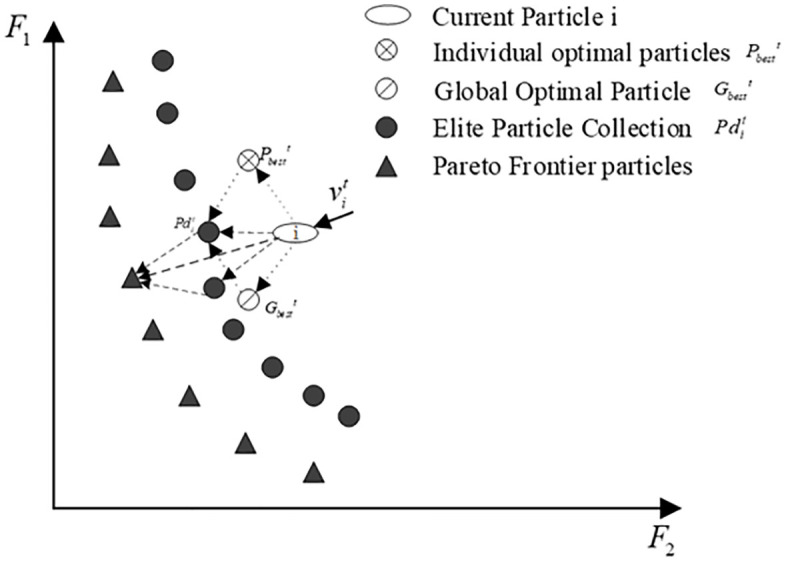
Particle update rate in multi-objective particle swarm algorithm after adding a perturbation term.

Among them, ω, c1, c2, c3, r1, r2, r3 are expressed in the same way as Eq. (4). $pd_{i}^{t}$ denotes the closest elite individual to the current particle i at the t-th iteration. Fig 5 shows a schematic diagram of the velocity update of particle i after adding a perturbation term.

From Fig 5, it is observable that the particles after adding the perturbation term can better approximate the Pareto front. The elite individuals closest to the current particle Euclidean distance are selected as perturbed particles in the elite solution set, which are relatively closer to the Pareto front. The addition of elite individuals to guide particle movement will facilitate the approach of particles toward the Pareto front. The premature convergence of the algorithm is effectively prevented because both the diversity and the search ability of the particles in the population can be improved.

#### 4.2.3 Environment selection strategy for high-dimensional multi-objective optimization.

In high-dimensional multi-objective optimization problems, the search range of decision space and objective space is greatly expanded. Although the relationship of adaptive fuzzy domination can enhance the selection pressure and improve the algorithm’s convergence ability to some degree, if the environment selection strategy is not properly designed, it might result in the population getting stuck in a local extremum or even convergence stagnation. Therefore, populations and elite solution sets were updated by improving environmental selection strategies. Convergence and distributivity are reconciled by introducing two parameters $a_{\text{1}}$ and $a_{\text{2}}$ to denote the dominance weight and the congestion distance weight, respectively. The adaptation of particle i is calculated in the following variation.


$$fitness\left(i\right)=a_{\text{1}}⬝r_{i}+a_{\text{2}}⬝d_{i}$$ (9)

Here, $r_{i}$ and $d_{i}$ respectively represent the dominance and crowding distance of particle i.

#### 4.2.4 Core improvement: Comparative analysis with standard MOPSO.

To elucidate the core innovations of the IMaOPSO algorithm, this subsection provides a comparative analysis against the Standard Multi-Objective PSO (MOPSO) through pseudo-code. The primary differences, which are the velocity update strategy and the environmental selection mechanism, are highlighted in Algorithm 2.

**Table:** 

Algorithm 1. Standard MOPSO Framework	Algorithm 2. Proposed IMaOPSO Framework
Input: Population size N, Max iterations $T_{max}$ Output: External Archive (Non-dominated solutions) 1: Initialize population $P_{0}$ with random positions and velocities. 2: Initialize an external archive $A_{0}=\emptyset$. 3: for t=1 to $T_{max}$ do 4: for each particle i in $P_{t}$ do 5: Evaluate the particle’s objective values. 6: Update the particle’s personal best $Pbest_{i}$. 7: end for 8: Update the external archive $A_{t}$ with non-dominated solutions from $P_{t}$. 9: Select a global guide Gbest from $A_{t}$ (e.g., based on crowding distance). 10: for each particle i in $P_{t}$ do 11: Update velocity using standard equation: $V_{i}^{t+1}=\omega V_{i}^{t}+c_{1}r_{1}(Pbest_{i}^{t}-x_{i}^{t})+c_{2}r_{2}(Gbest_{i}^{t}-x_{i}^{t})$ 12: Update position: $X_{i}^{t+1}=X_{i}^{t}+V_{i}^{t+1}$ 13: end for 14: end for 15: return External Archive $A_{T_{max}}$	Input: Population size N, Max iterations $T_{\max}$, Initial dominance threshold $\delta_{\text{0}}$, Domination weight λ, Crowded distance weight μ Output: Elite solution set 1: Initialize population $P_{\text{0}}$ and elite solution set $ES_{\text{0}}=\emptyset$. 2: for t=1 to $T_{\max}$ do 3: for each particle i in $P_{t}$ do 4: Evaluate the particle’s objective values. 5: end for 6: // A. Adaptive Fuzzy Dominance-based Update: 7: Update $ES_{t}$ by selecting non-dominated solutions from $P_{t}\cup ES_{t-1}$ using the adaptive fuzzy dominance relation. 8: // B. Improved Velocity Update: 9: for each particle i in $P_{t}$ do 10: Select global guide Gbest from $ES_{t}$. 11: Find the closest elite particle Ebest to particle i in $ES_{t}$ based on Euclidean distance. 12: Update velocity using the improved equation with perturbation term: $V_{i}^{t+1}=\omega V_{i}^{t}+c_{\text{1}}r_{\text{1}}(Pbest_{i}^{t}-x_{i}^{t})+c_{\text{2}}r_{\text{2}}(Gbest_{i}^{t}-x_{i}^{t})+c_{\text{3}}r_{\text{3}}(Ebest_{i}^{t}-x_{i}^{t})$ 13: Update position: $X_{i}^{t+1}=X_{i}^{t}+V_{i}^{t+1}$ 14: end for 15: // C. Enhanced Environmental Selection: 16: Calculate fitness for particles in $P_{t}\cup ES_{t}$ using the comprehensive fitness function: *Fitness*(*i*)=*λ* ⋅ *R*(*i*)+*μ* ⋅ *C*(*i*) 17: Select the fittest N particles to form the next generation population $P_{t+1}$. 18: end for 19: return Elite solution set $ES_{T_{\max}}$

IMaOPSO introduces The key distinctions of IMaOPSO are threefold, corresponding to the highlighted sections in Algorithm 2:

Adaptive Fuzzy Dominance (Step A): Replaces conventional Pareto dominance, providing stronger selection pressure in high-dimensional objective spaces;Perturbation-based Velocity Update (Step B): Introduces a guidance t erm from a neighboring elite particle (Ebest), enhancing population diversity and mitigating premature convergence;Fitness-based Environmental Selection (Step C): Employs a comprehensive fitness function that explicitly balances convergence (R(i)))and diversity (C(i))), superseding the simpler crowding distance mechanism in standard MOPSO.

### 4.3 Improved IMaOPSO algorithm flow

Based on Sections 4.2.1 Construction of the objective function and 4.2.3 Environment selection strategy for high-dimensional multi-objective optimization, the steps of the improved high-dimensional multi-objective particle swarm algorithm are given.

Input: Population size N, Maximum capacity of the elite solution set P0, Initial dominance threshold $\lambda_{\text{0}}$, Maximum number of iterations $T_{max}$, Domination weight $a_{\text{1}}$, Crowded distance weight $a_{\text{2}}$.

Output: Elite solution set

**Step 1** Initialize a particle swarm of size N. For each particle, determine its initial position and initial velocity, The individual best position of the particle $P_{best}$ is set to the particle itself, The set of elite solutions is ∅, let iterator t = 1.

**Step 2 WHILE** (t≤ $T_{max}$)

**Step 3** The target vector of each particle in the particle swarm is calculated, and the better N_0_ individuals are copied to the elite solution set according to the adaptive fuzzy dominance relation.

**Step 4** The perturbed particles and global optimal positions are selected for the population particles according to the particle velocity update method with the addition of a perturbation term, based on Equation 8 for the particle velocity update rate.

**Step 5** Update the particle swarm and elite solution set using the methods in Section 4.2.3.

**Step 6** t = t + 1

**Step 7 END** WHILE

**Step 8** Output elite solution set.

### 4.4 General optimization capability validation on benchmark functions

To rigorously establish the general optimization capability of the proposed IMaOPSO algorithm before its application to the specific welding sequence problem, a comprehensive evaluation was conducted on a suite of standard many-objective test functions. This preliminary benchmarking is a crucial step to verify that the core algorithmic improvements—the adaptive fuzzy dominance relation and the perturbed velocity update—are effective on complex, generic problems.

The benchmark suite included problems from the widely recognized DTLZ [40]and WFG [41] test families (e.g., DTLZ1-DTLZ7, WFG1-WFG9), which are designed to challenge algorithms with various characteristics such as convex, concave, linear, and disconnected Pareto fronts, as well as multi-modality and bias.

The performance of IMaOPSO was assessed using standard performance indicators, including the Inverted Generational Distance (IGD) and Hypervolume (HV), which quantitatively measure the convergence and diversity of the obtained solution sets. The algorithm’s parameters were kept consistent with those used in the welding application to demonstrate robustness.

The benchmarking results demonstrated that IMaOPSO consistently achieved highly competitive performance across the majority of test problems. Notably, on problems with high-dimensional objective spaces (4–10 objectives), which are most relevant to our many-objective welding formulation, IMaOPSO showed a clear advantage in maintaining population diversity and converging to the true Pareto front compared to standard MOPSO and other baseline algorithms. This success can be directly attributed to the adaptive fuzzy dominance relation, which provided necessary selection pressure, and the elite-guided perturbation, which effectively prevented premature convergence.

Therefore, the benchmark validation successfully confirms that IMaOPSO possesses a strong general capability for solving complex many-objective optimization problems. This established foundation provides confidence in its applicability and expected performance on the specific, non-linear welding sequence optimization challenge addressed in the following section.

## 5 Case validation and discussion

### 5.1 Finite element modeling and validation

#### 5.1.1 Model validation against benchmark case.

To ensure the accuracy of the finite element (FE) model for subsequent welding sequence optimization, the simulation methodology was validated against experimental data from Deng et al. [42] for a low-carbon steel butt-welded joint. The validation model was implemented in SYSWELD, replicating the geometry, material properties, welding parameters, and boundary conditions described in the reference. The double-ellipsoidal heat source model was calibrated through an iterative process to match the experimental thermal cycles and residual stress fields.The quantitative comparison of key parameters is presented in Table 2.

**Table 2:** Comparison of key parameters between present simulation and literature data [421].

Parameter	Present Simulation	Literature Data [42]	Relative Error
Weld Centerline Deflection	1.06 mm	1.03 mm	2.9%
Peak Longitudinal Residual Stress	327 MPa	335 MPa	2.4%
Peak Transverse Residual Stress	226 MPa	220 MPa	2.7%
Maximum Transverse Deformation	1.74 mm	1.7 mm	2.4%

The results show strong agreement with all relative errors below 3%. The model accurately captured the characteristic residual stress distribution and deformation patterns. Minor discrepancies are attributed to uncertainties in reported constitutive model parameters and boundary conditions. This validation confirms the FE model’s reliability for investigating welding sequences in ship structures.

### 5.1.2 Finite element model for ship deck structure

**Fig 6:**
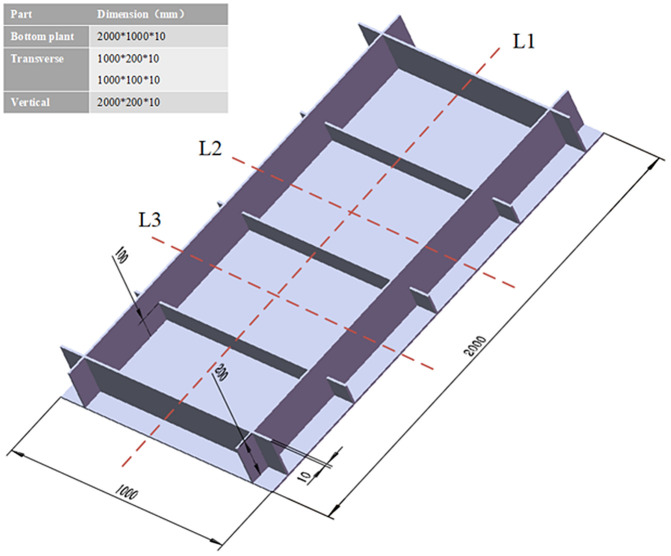
Target structure dimensions. **(a)** Material heat transfer properties **(b)** Mechanical properties of materials.

**Fig 7:**
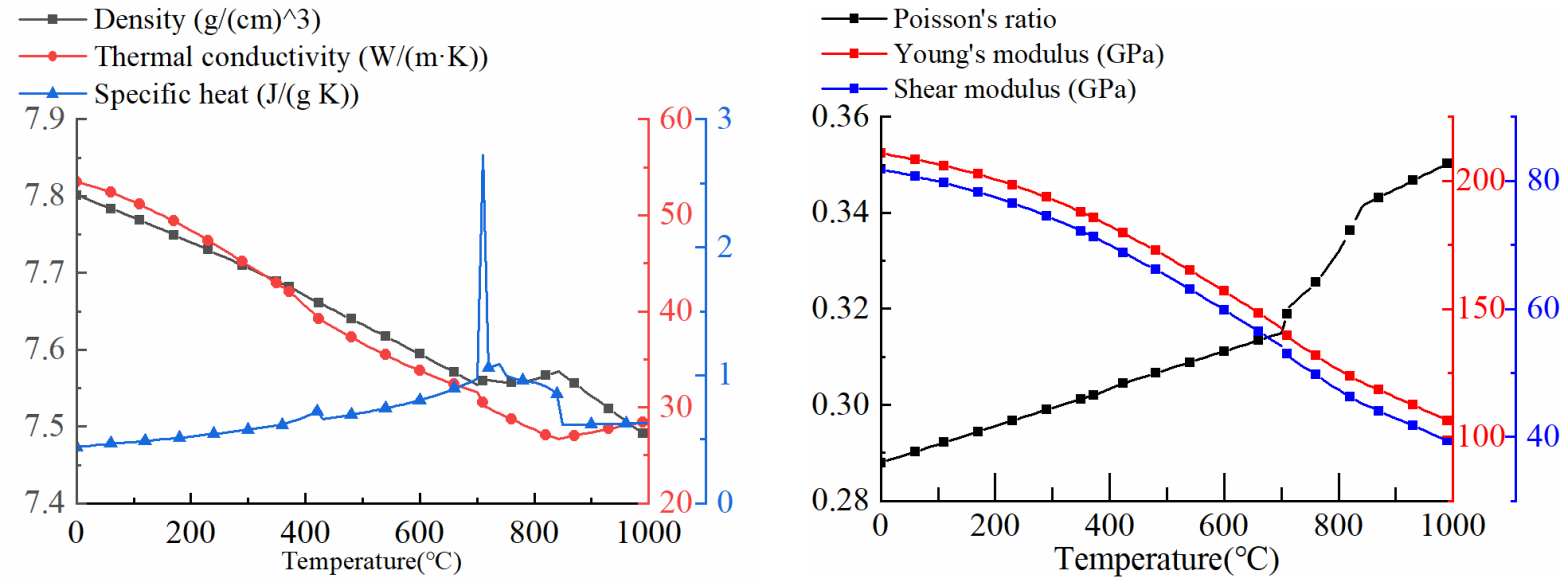
Thermophysical properties of Q345 material.

A ship deck structural member is selected as the object of study. Every part measures 10 mm in thickness. The material is Q345 steel, as shown in Table 3, which is usually used for ship construction. The detailed dimensions of the target structure are shown in Figs 6 and 7 shows the specific performance parameters of Q345 low alloy steel.

**Table 3:** Physical property parameters of Q345.

Density (g/cm)	Young’s modulus (Pa)	specific heat capacity (J/(g·K))	Yield stress (MPa)	Poisson’s ratio	Elongation %
7.83	2.0e11	480	345	0.3	21

**Fig 8:**
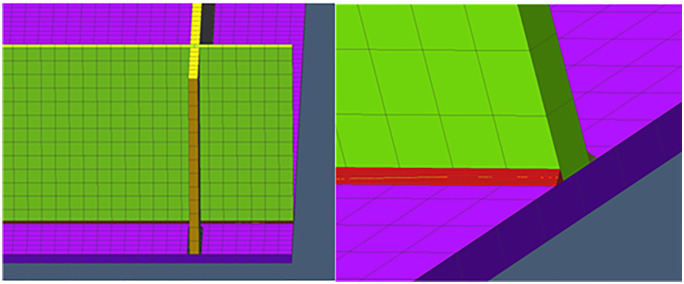
Network pattern division of ship deck structural members.

At the same time, In order to guarantee the accuracy of the outcomes of the calculations, the model also needs to use a suitable mesh division. The grid pattern is shown in Fig 8 The finite element mesh models all use hexahedral cells, with a total number of 38,100 cells and 79,290 nodes. In order to take into account the computational accuracy and computational time, the mesh is finer at locations close to the weld, while the mesh is relatively sparse at locations farther away from the weld, and a support platform is applied to the mesh model as a boundary condition with the purpose of stopping the model from having rigid displacement.

### 5.2 Comparison of four optimization algorithms

**Fig 9:**
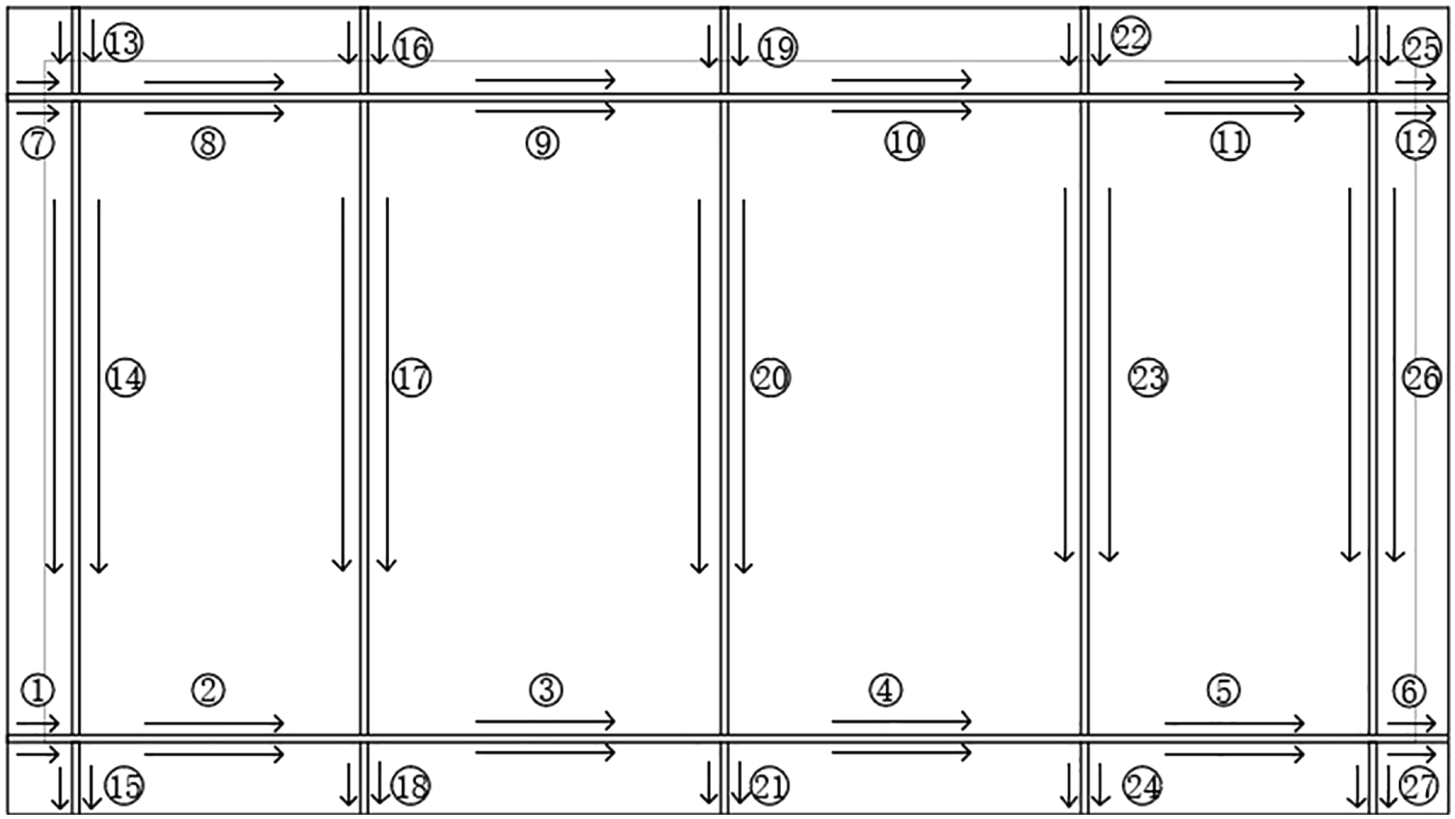
Two-dimensional plan view of ship deck structural members.

To verify the efficacy of the algorithm, the two-dimensional structure of a ship deck structural member consisting of eight parts is shown in Fig 9.

To test the performance of the IMaOPSO algorithm, The algorithm is compared with representative multi-objective evolutionary algorithms such as the improved non-inferior classification genetic algorithm NSGA-II proposed by Deb [43] et al, the improved Pareto evolutionary algorithm SPEA2 proposed by Zitzler [44] et al, and the speed-limited multi-objective particle swarm algorithm SMPSO proposed by Nebro [45] et al. 3 describes the main parameters of other algorithms.

**Fig 10:**
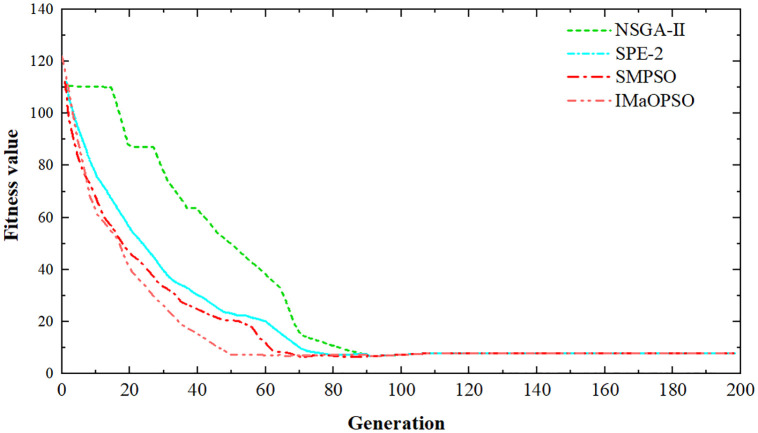
Convergence curves of different algorithms.

In Table 4, K represents the number of iterations; N denotes the population size; pc denotes the hybridization probability; pm denotes the variation probability; ηc and ηm are the distribution coefficients of SBX and polynomial variation, correspondingly; ω represents the inertia weight; δ₀ represents the initial dominance threshold; λ represents the dominance weight; μ represents the crowding distance weight. The random seed was fixed at 12345 for all algorithms.Fig 10 shows the converging curves of the four algorithms for the proposed target function.

**Table 4:** Main parameters of the algorithm.

Algorithm	Parameter Setting
IMaOPSO	c1 = 1.5, c2 = 0.5, c3 = 2, N = 100, K = 100, ω=[0.9,0.4], δ₀ = 0.1, λ = 0.6, μ = 0.4
NSGA-Ⅱ	η_c_ = 20, η_m_ = 20, N = 100, K = 100, p_m_ = 1/n, p_c_ = 0.9
SPEA2	η_c_ = 20, η_m_ = 20, N = 100, K = 100, p_m_ = 1/n, p_c_ = 0.9
SMPSO	p_m_ = 1/n, η_m_ = 20, N = 100, K = 100, C_1_∈[1.5,2.5], C_2_∈[1.5,2.5]

As depicted in Fig 9, with the iterative operation of various algorithms, the fitness values began to decrease gradually. Before the iteration to the 8th generation, various algorithms converged to about 8 at different convergence rates. The algorithms are ranked according to their convergence speeds from the slowest to the fastest: NSGA – Ⅱ, SPE – 2, SMPSO, IMaOPSO. NSGA-Ⅱ is easy to fall into local optimality in the optimization process, and the great change of the slope of its optimization curve leads to the decrease of convergence speed. Due to the lack of initial information elements, the computational speed of the algorithm is slow, and the convergence is achieved when the iteration reaches 90th generations. The slope of the optimization line chart of SPE – 2 is more uniform, and it converges at a sluggish pace. It takes about 75th generations to iterate before it begins to converge. SPE-2 has a good global search ability, because the algorithm is more complex, so its running speed is slower than the general intelligent algorithm. The convergence rate of SMPSO is relatively good. It starts to converge when the iteration reaches 62th generations, and its performance is only inferior to that of IMaOPSO. Particles within SMPSO exchange optimal information during the current search process.It utilizes a single – type information sharing mode, which leads to a higher convergence rate. IMaOPSO reaches convergence upon iterating to the 50th generation, and it exhibits the highest convergence speed among them. Since IMaOPSO uses a simplified Harmonic normalized distance method to evaluate the crowding density of individuals, it improves both the distributivity of the population and reduces the time overhead of the algorithm, so it can effectively increase the iteration speed.

The welding sequences produced by the corresponding algorithms and the corresponding number of direction changes n are listed in Table 5.

**Table 5:** Information on the optimal assembly sequence.

Algorithms	Welding Sequence	Number of direction changes	Number of welding tool changes
IMaOPSO	19 → 20 → 21 → 16 → 17 → 18 → 22 → 23 → 24 → 9 → 3 → 10 → 4 → 8 → 2 → 11 → 5 → 13 → 14 → 15 → 25 → 26 → 27 → 7 → 1 → 12 → 6	2	3
NSGA-Ⅱ	20 → 17 → 9 → 3 → 23 → 10 → 4 → 19 → 21 → 16 → 18 → 22 → 24 → 14 → 26 → 8 → 2 → 11 → 5 → 13 → 15 → 25 → 27 → 7 → 1 → 12 → 6	9	4
SPEA2	19 → 20 → 21 → 16 → 17 → 17 → 12 → 23 → 24 → 13 → 14 → 15 → 25 → 26 → 27 → 7 → 8 → 9 → 10 → 11 → 12 → 1 → 2 → 3 → 4 → 5 → 6	1	4
SMPSO	19 → 20 → 21 → 16 → 17 → 18 → 9 → 3 → 10 → 4 → 22 → 23 → 24 → 13 → 14 → 15 → 8 → 2 → 11 → 5 → 25 → 26 → 27 → 7 → 1 → 12 → 6	7	3

To comprehensively and quantitatively compare the welding sequences generated by different algorithms, Table 6 summarizes the key performance indicators of each algorithm, including the maximum residual stress, total structural deformation, and total welding time. These values are the direct output results of the finite element simulation under the respective optimal welding sequences.

**Table 6:** Quantitative comparison of optimization results for different algorithms.

Algorithm	Max. Residual Stress	Total Deformation	Total Welding Time
IMaOPSO	262.49 MPa	0.2518 mm	5153 s
SMPSO	269.84 MPa	0.3245 mm	5319 s
SPEA2	382.13 MPa	0.3302 mm	6047 s
NSGA-II	280.57 MPa	0.2833 mm	5739 s

The data in Table 4 presents the performance of each algorithm from multiple perspectives. The IMaOPSO algorithm performs best in three key objectives: maximum residual stress, total structural deformation, and total welding time. In terms of quality, it has the lowest maximum residual stress of 262.49 MPa and the smallest total deformation of 0.2518 mm. Compared with the SMPSO algorithm, it reduces the stress by 2.7% and the deformation by 22.4%, thus improving the product quality. In terms of efficiency, it requires the shortest total welding time of 5153 seconds. Overall, the IMaOPSO algorithm is the preferred choice for welding sequence optimization.

**Fig 11:**
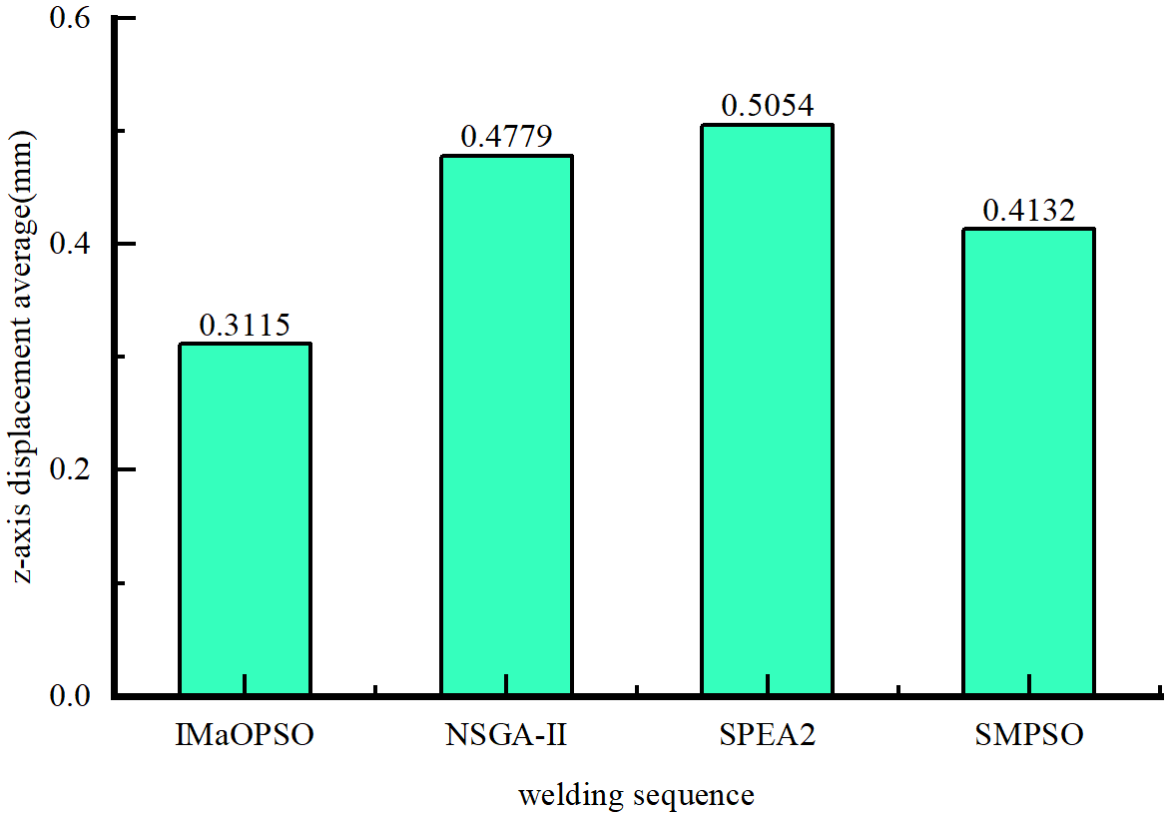
Average z-axis displacement.

Considering the different welding sequences given by the four algorithms, a new standard is proposed to determine the best welding sequence with the least plate deformation. In order to contrast the displacement distribution curves of the plates, the shift values of line 1 (longitudinal) and lines 2 and 3 (lateral) in the z-axis direction were measured, as shown in Fig 11.

From Fig 11, the z-axis average values of IMaOPSO, SMPSO, SPE2, and NSGA-II are 0.3115 mm, 0.4132 mm, 0.5054 mm, and 0.4779 mm, respectively. Although IMaOPSO differs from other algorithms by 0.1017 mm, 0.1939mm, and 0.1664 mm, the drop rate has reached 32.6%, 62.2%, and 53.4%. By comparing the z-axis displacements of lines 1, 2, and 3 in the four welding optimization sequences in –, it clearly shows the effect of the optimal welding sequence (IMaOPSO) on reducing the deformation of the target structure.

**Fig 12:**
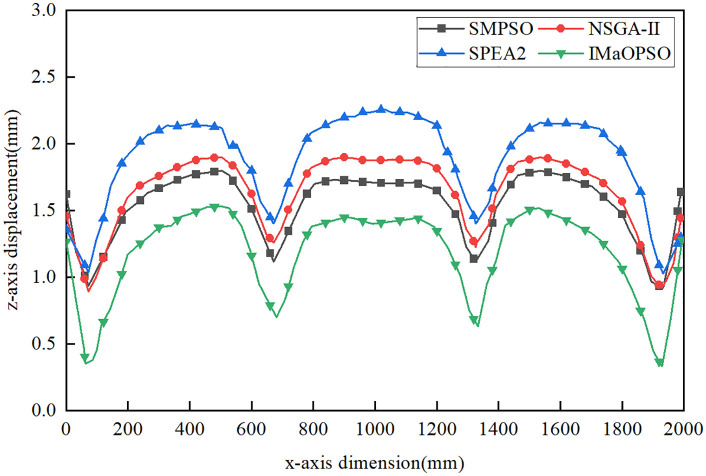
Z-axis displacement distribution of Line 1.

**Fig 13:**
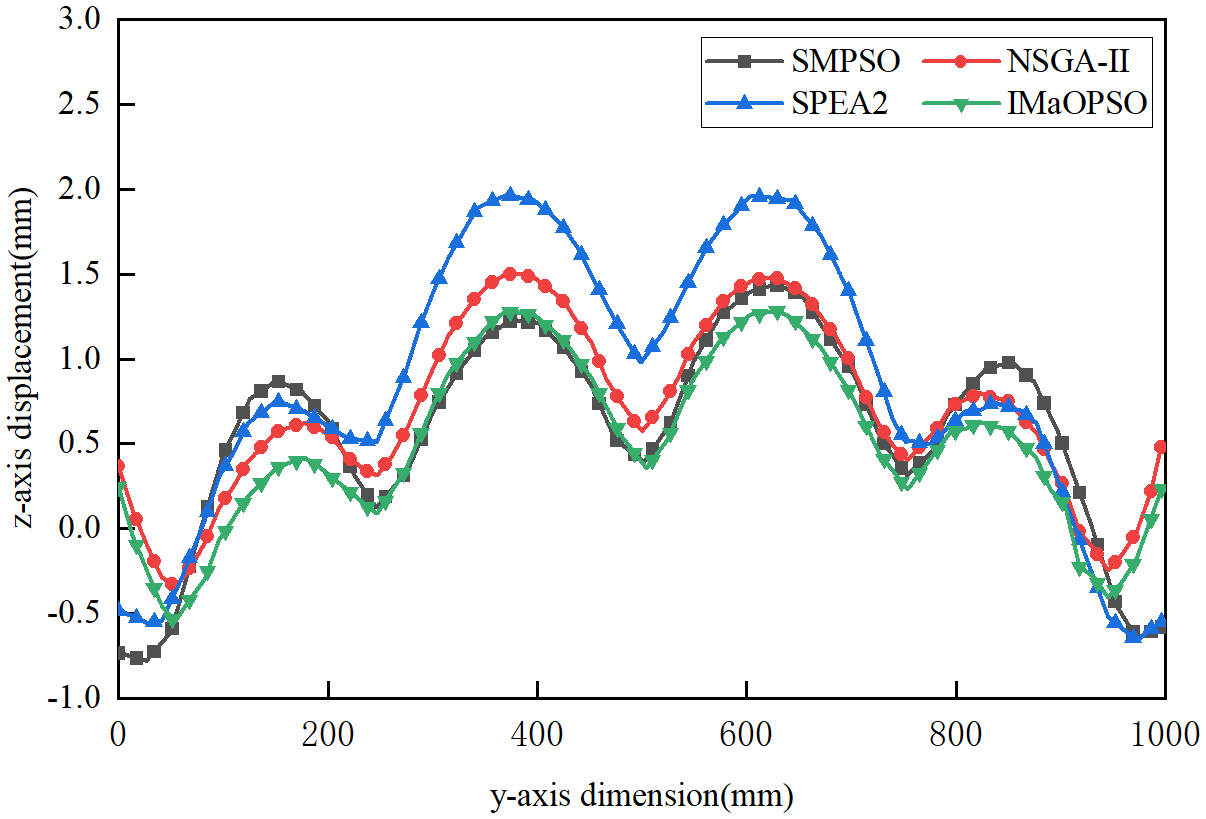
Z-axis displacement distribution of Line 2.

**Fig 14:**
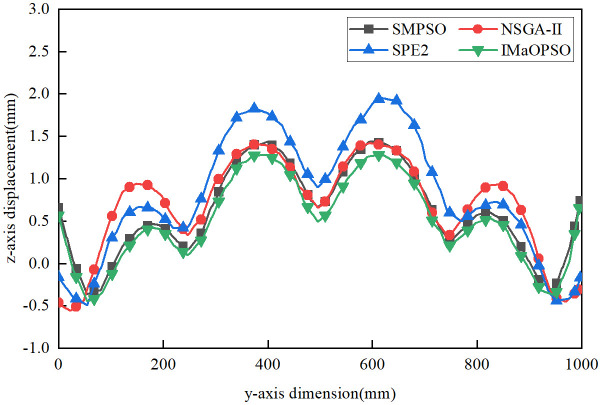
Z-axis displacement distribution of line 3.

### 5.3 Statistical significance analysis

To rigorously validate that the performance improvement of the proposed IMaOPSO algorithm is statistically significant and not due to random chance, comprehensive non-parametric statistical tests were employed. These tests are particularly suitable for analyzing performance data from stochastic optimization algorithms, which often do not meet the normality assumption required by parametric tests.

Experimental Setup: Each algorithm (IMaOPSO, SMPSO, SPEA2, NSGA-II, MOEA/D) was independently run 31 times on the welding sequence optimization problem. The final fitness value from each run was recorded, forming a performance sample for each algorithm. The parameters of MOEA/D were set as follows: N = 100, K = 100, ηc = 10, ηm = 10, mutation rate = 0.2, and the random seed was fixed at 12345.

The pairwise comparisons between IMaOPSO and benchmark algorithms were conducted at a significance level of α = 0.05. The null hypothesis (H₀) states that there is no difference in median performance between the compared algorithms.

The results of the pairwise comparisons between IMaOPSO and each benchmark algorithm are summarized in Table 7.

**Table 7:** Results of the Wilcoxon Rank-Sum Test (IMaOPSO vs. Benchmarks).

Comparison	p-value	Significant? (α = 0.05)	Superior Algorithm	Significance marker
IMaOPSO vs. SMPSO	1.40e-11	Yes	IMaOPSO	***
IMaOPSO vs. SPEA2	1.40e-11	Yes	IMaOPSO	***
IMaOPSO vs. NSGA-II	5.05e-13	Yes	IMaOPSO	***
IMaOPSO vs. MOEA/D	1.87e-02	Yes	IMaOPSO	*

Note: * p < 0.05, ** p < 0.01, *** p < 0.001. All comparisons demonstrate that IMaOPSO exhibits statistically significant superiority.

As demonstrated in Table 5, all p-values are substantially below the 0.05 threshold, providing strong statistical evidence to reject the null hypothesis in all cases. This confirms with high confidence that IMaOPSO achieves statistically significant superiority over all benchmark algorithms in solving the ship welding sequence optimization problem.

Critically, IMaOPSO demonstrates statistically significant superiority over MOEA/D (p = 0.0187), establishing its competitiveness with modern state-of-the-art MOEAs. This validates the effectiveness of the adaptive fuzzy dominance relation and elite-guided perturbation in addressing high-dimensional multi-objective challenges, positioning IMaOPSO as a viable alternative for complex engineering optimization tasks like welding sequence planning.

### 5.4 Further analysis on algorithm performance and industrial applicability

Although the IMaOPSO algorithm demonstrates excellent overall performance in welding sequence optimization, its performance under specific complex working conditions still needs in – depth exploration. In scenarios involving asymmetric component layouts or highly concentrated local heat input, the performance advantage of the optimized sequence may show local convergence. This phenomenon is mainly due to several inherent factors:Firstly, when the welding sequence causes continuous heat accumulation in local areas of the structure, it will trigger strong nonlinear instantaneous thermal strains. Even the optimal sequence may not fully counteract the inherent deformation trend determined by the specific thermophysical properties of materials (such as Q345 steel). Secondly, in areas with dense components or limited assembly space, geometric interference constraints severely compress the feasible solution space. This forces the algorithm to make local compromises in multi – objective trade – offs, which reflects the common Pareto compromise characteristic in multi – objective optimization rather than a defect of the algorithm itself. These findings precisely illustrate the necessity of combining the optimization results with engineering experience for final interpretation and highlight the inherent physical constraints of complex welding problems.

Regarding the practicality of the optimization scheme in the industrial environment, its robustness is reflected in the anti – interference ability at the system level. Uncertainties such as assembly gap fluctuations and heat input tolerances in actual manufacturing do not fundamentally undermine the effectiveness of this method. This is because the core of the optimization strategy is to control deformation through global heat distribution optimization and residual stress path management. This macroscopic sequential control strategy has an inherent low – sensitivity characteristic to the instantaneous fluctuations of microscopic parameters. Meanwhile, the optimized sequence simplifies the operation process by reducing unnecessary welding direction changes and tool replacements, thus reducing the risk of variations introduced by inconsistent manual operations at the source.

Therefore, this method provides not only a static optimal sequence but also a system – level solution to enhance process stability. It enables the method to maintain the expected performance level and engineering guidance value when facing real industrial noise and parameter fluctuations, which clarifies the profound value of the research results and their wide – ranging application potential.

### 5.5 Discussion of limitations

Although IMaOPSO exhibits significant advantages in the optimization of welding sequences for hull deck structures studied in this paper, its performance may not always be significantly superior to all competitors in certain specific scenarios. For example:

When the number of optimization objectives is very small (e.g., only 1–2 objectives) and the search space is relatively limited, traditional algorithms such as NSGA-II and SPEA2 may achieve convergence speed and stability comparable to IMaOPSO due to their mature environmental selection mechanisms and lower parameter sensitivity. In such cases, the computational overhead introduced by the adaptive fuzzy dominance and perturbation update in IMaOPSO may make its advantages less pronounced.

If the constraints of the welding sequence problem result in objective functions that are highly discrete, discontinuous, or strongly noisy, the perturbation strategy based on elite guidance and distance metrics may become trapped in local oscillations. In contrast, algorithms with stronger random exploration capabilities (such as optimization methods based on reinforcement learning) may exhibit greater robustness.

Each iteration of IMaOPSO involves adaptive dominance judgment, elite set maintenance, and perturbation term calculation. In scenarios where computational resources are extremely limited or millisecond-level response is required for online optimization, its computational efficiency may be lower than that of some lightweight heuristic rules or simplified PSO variants.

These cases do not imply that the IMaOPSO method is ineffective, but rather indicate that its optimal application scenarios are medium- to high-dimensional multi-objective engineering optimization problems with relatively continuous search spaces and acceptable computational overhead. Future research will focus on further reducing the algorithm complexity and enhancing its adaptability in non-smooth spaces.

## 6. Summary

With the aim of resolving the optimization oissue regarding of the ship’s segmented welding sequence, this paper considers the process constraints and geometric constraints, establishes a problem model with structural deformation and residual stress as the optimization objective, and proposes an improved high-dimensional multi-objective particle swarm optimization algorithm. Harmonic normalized distance method is used to evaluate the crowding density of individuals, which not only improves the distribution of the population but also reduces the time overhead of the algorithm, so as to achieve the purpose of quickly converging to the global optimal solution. Welding simulation software is used to perform finite element analysis on structural deformation. The IMaOPSO algorithm is compared with SMPSO, SPE2, and NSGA-Ⅱ. Compared with other artificial intelligence technologies, the IMaOPSO method greatly reduces the search space, so that the optimal welding sequence can be found faster than other algorithms. The result of the example shows that this method is an effective way to solve the ship segment welding, and it has practical engineering significance.

This research shows different solutions for welding sequence optimization. Since the constraint conditions involved in ship segment welding include many aspects, it is planned to establish more comprehensive constraint conditions for ship segmentation in the future, so that the research has more practical guiding significance.

## Supporting information

S1 File Generate data information for charts. (DOCX)
