# Supplementary material for: Improved many-objective particle swarm optimization based welding sequence optimization research
Source: PLoS One. 2026 Mar 5;21(3):e0343554. doi: 10.1371/journal.pone.0343554 (PMC12962489; doi:10.1371/journal.pone.0343554)
Supplement: S1 File — (DOCX) [file pone.0343554.s001.docx]

**The data in Figure 7**

| Temperature(℃) | Density(g/cm³) | Thermal conductivity  (W/(m·K)) | Specific heat  (J/(g·K)) |
| --- | --- | --- | --- |
| 0 | 7.8 | 7.8 | 7.45 |
| 100 | 7.78 | 7.75 | 7.46 |
| 200 | 7.75 | 7.7 | 7.47 |
| 300 | 7.72 | 7.65 | 7.48 |
| 400 | 7.68 | 7.6 | 7.51 |
| 500 | 7.65 | 7.55 | 7.52 |
| 600 | 7.62 | 7.5 | 7.53 |
| 700 | 7.58 | 7.45 | 7.55 |
| 800 | 7.55 | 7.4 | 7.52 |
| 900 | 7.52 | 7.35 | 7.5 |
| 1000 | 7.5 | 7.3 | 7.5 |

| Temperature(℃) | Poisson’s ratio | Young’s modulus  (GPa) | Shear modulus  (GPa) |
| --- | --- | --- | --- |
| 0 | 0.29 | 0.35 | 0.35 |
| 100 | 0.295 | 0.345 | 0.345 |
| 200 | 0.3 | 0.34 | 0.34 |
| 300 | 0.305 | 0.335 | 0.335 |
| 400 | 0.31 | 0.33 | 0.33 |
| 500 | 0.315 | 0.325 | 0.325 |
| 600 | 0.32 | 0.32 | 0.32 |
| 700 | 0.32 | 0.315 | 0.31 |
| 800 | 0.335 | 0.31 | 0.305 |
| 900 | 0.345 | 0.305 | 0.295 |
| 1000 | 0.35 | 0.295 | 0.29 |

**The data in Figure 11**

| Algorithm | Z-axis displacement average(mm) |
| --- | --- |
| IMaOPSO | 0.3115 |
| NSGA-II | 0.4779 |
| SPEA2 | 0.5054 |
| SMPSO | 0.4132 |

**The data in Figure 12**

| x-axis (mm) | SMPSO | NSGA-II | SPEA2 | IMaOPSO |
| --- | --- | --- | --- | --- |
| 0 | 1.6 | 1 | 1.4 | 1.3 |
| 50 | 1.2 | 0.9 | 1.1 | 0.4 |
| 100 | 0.9 | 0.9 | 1 | 0.7 |
| 150 | 1.2 | 1.2 | 1.5 | 1 |
| 200 | 1.5 | 1.5 | 2 | 1.3 |
| 250 | 1.8 | 1.8 | 2.2 | 1.5 |
| 300 | 2 | 1.9 | 2.3 | 1.5 |
| 350 | 1.8 | 1.8 | 2 | 1.2 |
| 400 | 1.5 | 1.5 | 1.4 | 0.8 |
| 450 | 1.1 | 1.2 | 1 | 0.7 |
| 500 | 1.2 | 1.5 | 1.8 | 1 |
| 550 | 1.5 | 1.8 | 2.2 | 1.3 |
| 600 | 1.8 | 1.9 | 2.3 | 1.5 |
| 650 | 2 | 2 | 2.3 | 1.5 |
| 700 | 1.8 | 1.9 | 2.2 | 1.3 |
| 750 | 1.5 | 1.8 | 2 | 1 |
| 800 | 1.2 | 1.5 | 1.8 | 1.3 |
| 850 | 1.5 | 1.8 | 2.2 | 1.5 |
| 900 | 1.8 | 1.9 | 2.3 | 1.5 |
| 950 | 2 | 2 | 2.3 | 1.5 |
| 1000 | 1.8 | 1.9 | 2.2 | 1.3 |
| 1050 | 1.5 | 1.8 | 2 | 1 |
| 1100 | 1.2 | 1.5 | 1.8 | 1.3 |
| 1150 | 1.5 | 1.8 | 2.2 | 1.5 |
| 1200 | 1.8 | 1.9 | 2.3 | 1.5 |
| 1250 | 2 | 2 | 2.3 | 1.5 |
| 1300 | 1.8 | 1.9 | 2.2 | 1.3 |
| 1350 | 1.5 | 1.8 | 2 | 1 |
| 1400 | 1.1 | 1.2 | 1.4 | 0.6 |
| 1450 | 1.2 | 1.5 | 1.8 | 1 |
| 1500 | 1.5 | 1.8 | 2.2 | 1.3 |
| 1550 | 1.8 | 1.9 | 2.3 | 1.5 |
| 1600 | 2 | 1.9 | 2.3 | 1.5 |
| 1650 | 1.8 | 1.8 | 2 | 1.2 |
| 1700 | 1.5 | 1.5 | 1.8 | 1.3 |
| 1750 | 1.8 | 1.8 | 2.2 | 1.5 |
| 1800 | 2 | 1.9 | 2.3 | 1.5 |
| 1850 | 1.8 | 1.8 | 2 | 1.2 |
| 1900 | 1.5 | 1.5 | 1.4 | 0.9 |
| 1950 | 1.6 | 1.4 | 1.2 | 1 |
| 2000 | 1.6 | 1.4 | 1.3 | 1.1 |

**The data in Figure 13**

| y-axis | SMPSO | NSGA-II | SPEA2 | IMaOPSO |
| --- | --- | --- | --- | --- |
| 0 | -0.8 | 0.5 | -0.6 | -0.5 |
| 50 | -0.5 | 0 | -0.4 | -0.2 |
| 100 | -0.2 | 0.3 | 0 | 0.1 |
| 150 | 0.8 | 0.6 | 0.7 | 0.5 |
| 200 | 0.4 | 0.3 | 0.5 | 0.2 |
| 250 | 0.2 | 0.2 | 0.3 | 0.1 |
| 300 | 0.5 | 0.8 | 1.2 | 0.9 |
| 350 | 1 | 1.3 | 1.8 | 1.2 |
| 400 | 1.2 | 1.5 | 2 | 1.3 |
| 450 | 1 | 1.3 | 1.8 | 1.2 |
| 500 | 0.5 | 0.8 | 1.2 | 0.9 |
| 550 | 0.2 | 0.5 | 1 | 0.6 |
| 600 | 1.4 | 1.5 | 2 | 1.3 |
| 650 | 1.2 | 1.3 | 1.8 | 1.2 |
| 700 | 0.8 | 1 | 1.4 | 0.9 |
| 750 | 0.3 | 0.4 | 0.5 | 0.3 |
| 800 | 0.8 | 0.7 | 0.8 | 0.7 |
| 850 | 0.9 | 0.8 | 0.9 | 0.8 |
| 900 | 0.5 | 0.2 | -0.4 | -0.1 |
| 950 | -0.5 | -0.3 | -0.6 | -0.4 |
| 1000 | -0.6 | 0.5 | -0.7 | 0.3 |

**The data in Figure 14**

| y-axis (mm) | SMPSO | NSGA-II | SPE2 | IMaOPSO |
| --- | --- | --- | --- | --- |
| 0 | 0.7 | -0.5 | -0.3 | 0.6 |
| 50 | 0 | -0.4 | -0.5 | -0.4 |
| 100 | -0.1 | -0.1 | -0.2 | -0.5 |
| 150 | 0.3 | 0.8 | 0.5 | 0 |
| 200 | 0.4 | 0.9 | 0.6 | 0.4 |
| 250 | 0.3 | 0.7 | 0.4 | 0.2 |
| 300 | 0.6 | 1 | 1.3 | 0.7 |
| 350 | 0.9 | 1.3 | 1.7 | 1.1 |
| 400 | 1.2 | 1.4 | 1.9 | 1.3 |
| 450 | 1.1 | 1.3 | 1.7 | 1.2 |
| 500 | 0.6 | 1 | 1.3 | 0.7 |
| 550 | 0.3 | 0.8 | 1 | 0.5 |
| 600 | 1.4 | 1.4 | 1.9 | 1.3 |
| 650 | 1.2 | 1.3 | 1.7 | 1.2 |
| 700 | 0.9 | 1.1 | 1.4 | 0.9 |
| 750 | 0.4 | 0.5 | 0.6 | 0.3 |
| 800 | 0.3 | 0.3 | 0.4 | 0.2 |
| 850 | 0.6 | 0.9 | 0.7 | 0.4 |
| 900 | 0.4 | 0.7 | 0.5 | 0.2 |
| 950 | 0 | 0 | -0.2 | -0.4 |
| 1000 | 0.7 | -0.3 | -0.4 | 0.6 |

For the data in Table 7, we ran the IMaOPSO, SMPSO, SPEA2, NSGA-II, and MOEA/D algorithms independently 31 times under identical experimental conditions Record the fitness values obtained from each run as follows:

| Algorithm | fitness values |
| --- | --- |
| IMaOPSO | 20.0525554, 20.00054244, 20.24416709, 20.10981923, 20.69054186, 20.68306968,20.30776498, 20.59442043, 20.44799165, 20.37080763, 20.12288292, 20.27950679,20.1022213, 20.21938087, 20.16506252, 19.90919494, 20.03071528, 20.29589787,19.77736176, 20.3566365, 20.12276073, 19.90217904, 20.50293797, 20.34430343,20.36775767, 20.32759456, 20.21107334, 20.26271294, 19.96643018, 20.32245654,  19.98907551 |
| SMPSO | 24.703205, 23.931639, 24.060605, 24.334654, 23.619740, 23.928563,  24.154700, 24.543186, 23.821061, 24.521967, 24.698941, 23.508248,  24.264874, 24.350239, 23.640783, 23.958008, 24.677419, 24.534858,  24.260641, 23.886002, 24.446854, 23.654536, 24.514965, 24.105959,  24.291944, 24.454372, 24.040641, 24.594292, 24.524257, 24.366128,  23.845027 |
| SPEA2 | 22.478564, 22.938422, 21.458488, 22.962875, 22.466083, 22.421982,  22.597237, 23.067726, 22.253312, 22.677927, 23.128487, 22.676488,  22.888270, 22.886866, 22.753758, 22.431200, 22.528282, 22.507851,  22.750831, 22.850283, 22.793743, 22.107148, 23.281116, 22.376540,  22.889665, 22.277518, 22.817664, 22.991713, 22.982099, 22.553474,  22.728756 |
| NSGA-II | 21.89018046, 21.89018046, 21.89018046, 21.89018046, 21.89018046, 21.89018046,21.89018046, 21.89018046, 21.89018046, 21.89018046, 21.89018046, 21.89018046, 21.89018046, 21.89018046, 21.89018046, 21.89018046, 21.89018046, 21.89018046,21.89018046, 21.89018046, 21.89018046, 21.89018046, 21.89018046, 21.89018046, 21.89018046, 21.89018046, 21.89018046, 21.89018046, 21.89018046, 21.89018046,  21.89018046 |
| MOEA/D | 20.49717878, 20.13683424, 20.2198812, 20.24128785, 20.13868914, 20.44719732,20.2555349, 20.26159989, 20.39175304, 20.15665735,  20.4058533, 20.36283212,20.36732545, 20.44053521, 20.38979726, 20.48367746, 20.34247539, 20.22970415,20.51075463, 20.38724287, 20.33493699, 20.32311803, 20.29885292, 20.17233037,20.25726984, 20.36379038, 20.29213619, 20.40577038, 20.44814694, 20.37112689,  20.2358113 |

Wilcoxon rank sum test：

import numpy as np
from scipy import stats

imaopso_data = np.array([20.0525554, 20.00054244, 20.24416709, 20.10981923, 20.69054186, 20.68306968,
 20.30776498, 20.59442043, 20.44799165, 20.37080763, 20.12288292, 20.27950679,
 20.1022213, 20.21938087, 20.16506252, 19.90919494, 20.03071528, 20.29589787,
 19.77736176, 20.3566365, 20.12276073, 19.90217904, 20.50293797, 20.34430343,
 20.36775767, 20.32759456, 20.21107334, 20.26271294, 19.96643018, 20.32245654,
 19.98907551])

smpso_data = np.array([24.703205, 23.931639, 24.060605, 24.334654, 23.619740, 23.928563,
 24.154700, 24.543186, 23.821061, 24.521967, 24.698941, 23.508248,
 24.264874, 24.350239, 23.640783, 23.958008, 24.677419, 24.534858,
 24.260641, 23.886002, 24.446854, 23.654536, 24.514965, 24.105959,
 24.291944, 24.454372, 24.040641, 24.594292, 24.524257, 24.366128,
 23.845027])

spea2_data = np.array([22.478564, 22.938422, 21.458488, 22.962875, 22.466083, 22.421982,
 22.597237, 23.067726, 22.253312, 22.677927, 23.128487, 22.676488,
 22.888270, 22.886866, 22.753758, 22.431200, 22.528282, 22.507851,
 22.750831, 22.850283, 22.793743, 22.107148, 23.281116, 22.376540,
 22.889665, 22.277518, 22.817664, 22.991713, 22.982099, 22.553474,
 22.728756])

nsga2_data = np.array([21.89018046, 21.89018046, 21.89018046, 21.89018046, 21.89018046, 21.89018046,
 21.89018046, 21.89018046, 21.89018046, 21.89018046, 21.89018046, 21.89018046,
 21.89018046, 21.89018046, 21.89018046, 21.89018046, 21.89018046, 21.89018046,
 21.89018046, 21.89018046, 21.89018046, 21.89018046, 21.89018046, 21.89018046,
 21.89018046, 21.89018046, 21.89018046, 21.89018046, 21.89018046, 21.89018046,
 21.89018046])

moead_data = np.array([20.49717878, 20.13683424, 20.2198812, 20.24128785, 20.13868914, 20.44719732,
 20.2555349, 20.26159989, 20.39175304, 20.15665735, 20.4058533, 20.36283212,
 20.36732545, 20.44053521, 20.38979726, 20.48367746, 20.34247539, 20.22970415,
 20.51075463, 20.38724287, 20.33493699, 20.32311803, 20.29885292, 20.17233037,
 20.25726984, 20.36379038, 20.29213619, 20.40577038, 20.44814694, 20.37112689,
 20.2358113])


def perform_all_wilcoxon_tests(imaopso, smpso, spea2, nsga2, moead, alpha=0.05):

 algorithms = {
 'IMaOPSO': imaopso,
 'SMPSO': smpso,
 'SPEA2': spea2,
 'NSGA-II': nsga2,
 'MOEA/D': moead
 }

 algorithm_names = list(algorithms.keys())
 n_algorithms = len(algorithm_names)

 print("=" * 80)
 print("Wilcoxon Rank-Sum Test Results (All Pairwise Comparisons)")
 print("=" * 80)

 print("\nBasic Statistics:")
 print(f"{'Algorithm':<10} {'Mean':<12} {'Std':<12} {'Min':<12} {'Max':<12}")
 for name in algorithm_names:
 data = algorithms[name]
 print(f"{name:<10} {np.mean(data):<12.6f} {np.std(data):<12.6f} {np.min(data):<12.6f} {np.max(data):<12.6f}")

 results = []

 for i in range(n_algorithms):
 for j in range(i + 1, n_algorithms):
 algo1 = algorithm_names[i]
 algo2 = algorithm_names[j]
 data1 = algorithms[algo1]
 data2 = algorithms[algo2]

 stat, p_value = stats.mannwhitneyu(data1, data2, alternative='two-sided')

 mean1 = np.mean(data1)
 mean2 = np.mean(data2)

 if mean1 < mean2:
 superior = algo1
 else:
 superior = algo2

 significant = p_value < alpha

 results.append({
 'Algorithm1': algo1,
 'Algorithm2': algo2,
 'Statistic': stat,
 'p-value': p_value,
 'Significant': significant,
 'Superior': superior
 })

 print(f"\nPairwise Wilcoxon Rank-Sum Test Results (α = {alpha}):")
 print(f"{'Comparison':<20} {'Statistic':<12} {'p-value':<15} {'Significant':<12} {'Superior':<10}")
 print("-" * 75)

 for result in results:
 sig_symbol = "✓" if result['Significant'] else "✗"
 p_str = f"{result['p-value']:.2e}"
 print(
 f"{result['Algorithm1']} vs {result['Algorithm2']:<12} {result['Statistic']:<12.2f} {p_str:<15} {sig_symbol:<12} {result['Superior']:<10}")

 return results


def create_pvalue_matrix(imaopso, smpso, spea2, nsga2, moead):

 algorithms = {
 'IMaOPSO': imaopso,
 'SMPSO': smpso,
 'SPEA2': spea2,
 'NSGA-II': nsga2,
 'MOEA/D': moead
 }

 algorithm_names = list(algorithms.keys())
 n_algorithms = len(algorithm_names)

 pvalue_matrix = np.ones((n_algorithms, n_algorithms))

 for i in range(n_algorithms):
 for j in range(n_algorithms):
 if i != j:
 data1 = algorithms[algorithm_names[i]]
 data2 = algorithms[algorithm_names[j]]
 _, p_value = stats.mannwhitneyu(data1, data2, alternative='two-sided')
 pvalue_matrix[i, j] = p_value

 print("\n" + "=" * 60)
 print("P-value Matrix")
 print("=" * 60)

 print(f"{'':<10}", end="")
 for name in algorithm_names:
 print(f"{name:<12}", end="")
 print()

 for i, name1 in enumerate(algorithm_names):
 print(f"{name1:<10}", end="")
 for j, name2 in enumerate(algorithm_names):
 if i == j:
 print(f"{'--':<12}", end="")
 else:
 p_val = pvalue_matrix[i, j]
 if p_val < 0.001:
 print(f"{'<0.001':<12}", end="")
 else:
 print(f"{p_val:.3f}{'*' if p_val < 0.05 else '':<11}", end="")
 print()

 print("\n* indicates p-value < 0.05")


def perform_detailed_analysis(imaopso, smpso, spea2, nsga2, moead):

 algorithms = {
 'IMaOPSO': imaopso,
 'SMPSO': smpso,
 'SPEA2': spea2,
 'NSGA-II': nsga2,
 'MOEA/D': moead
 }

 print("\n" + "=" * 80)
 print("Detailed Performance Analysis")
 print("=" + "=" * 79)

 imaopso_mean = np.mean(imaopso)
 print(f"\nPerformance Improvement Relative to IMaOPSO (Mean = {imaopso_mean:.6f}):")
 print(f"{'Algorithm':<10} {'Mean':<12} {'Improvement':<15} {'Status':<10}")
 print("-" * 55)

 for name, data in algorithms.items():
 if name != 'IMaOPSO':
 other_mean = np.mean(data)
 if other_mean < imaopso_mean:
 improvement = ((imaopso_mean - other_mean) / imaopso_mean) * 100
 status = "Worse"
 else:
 improvement = ((other_mean - imaopso_mean) / other_mean) * 100
 status = "Better"

 print(f"{name:<10} {other_mean:<12.6f} {improvement:<15.2f}% {status:<10}")


if __name__ == "__main__":
 results = perform_all_wilcoxon_tests(imaopso_data, smpso_data, spea2_data, nsga2_data, moead_data)

 create_pvalue_matrix(imaopso_data, smpso_data, spea2_data, nsga2_data, moead_data)

 perform_detailed_analysis(imaopso_data, smpso_data, spea2_data, nsga2_data, moead_data)

 print("\n" + "=" * 80)
 print("Friedman Test (Overall Comparison)")
 print("=" + "=" * 79)

 friedman_stat, friedman_p = stats.friedmanchisquare(
 imaopso_data, smpso_data, spea2_data, nsga2_data, moead_data
 )
 print(f"Friedman statistic: {friedman_stat:.4f}")
 print(f"p-value: {friedman_p:.2e}")

 if friedman_p < 0.05:
 print("Conclusion: Significant differences exist among all algorithms (p < 0.05)")
 else:
 print("Conclusion: No significant differences among algorithms (p ≥ 0.05)")
 print("\n" + "=" * 40)
 print("Algorithm Ranking (from best to worst)")
 print("=" * 40)
 algorithm_means = {
 'IMaOPSO': np.mean(imaopso_data),
 'MOEA/D': np.mean(moead_data),
 'NSGA-II': np.mean(nsga2_data),
 'SPEA2': np.mean(spea2_data),
 'SMPSO': np.mean(smpso_data)
 }
 sorted_algorithms = sorted(algorithm_means.items(), key=lambda x: x[1])
 for rank, (algo, mean_val) in enumerate(sorted_algorithms, 1):
 print(f"{rank}. {algo:<10}: {mean_val:.6f}")
